# Donor lung derived myeloid and plasmacytoid dendritic cells differentially regulate T cell proliferation and cytokine production

**DOI:** 10.1186/1465-9921-13-25

**Published:** 2012-03-20

**Authors:** Heather L Benson, Hidemi Suzuki, Jeremy Lott, Amanda Jo Fisher, Crystal Walline, Kathleen M Heidler, Randy Brutkiewicz, Janice S Blum, David S Wilkes

**Affiliations:** 1 Department of Medicine, Microbiology and Immunology, Indiana University School of Medicine, Indianapolis, IN 46202, USA; 2 Center for Immunobiology, Indiana University School of Medicine, Indianapolis, IN 46202, USA; 3 Department of Thoracic Surgery, Chiba University, Chiba, Japan; 4 Department of Anesthesia, Indiana University School of Medicine, Indianapolis, IN 46202, USA

**Keywords:** Lung transplantation, dendritic cells, mouse, T cell activation

## Abstract

**Background:**

Direct allorecognition, i.e., donor lung-derived dendritic cells (DCs) stimulating recipient-derived T lymphocytes, is believed to be the key mechanism of lung allograft rejection. Myeloid (cDCs) and plasmacytoid (pDCs) are believed to have differential effects on T cell activation. However, the roles of each DC type on T cell activation and rejection pathology post lung transplantation are unknown.

**Methods:**

Using transgenic mice and antibody depletion techniques, either or both cell types were depleted in lungs of donor BALB/c mice (H-2^d^) prior to transplanting into C57BL/6 mice (H-2^b^), followed by an assessment of rejection pathology, and pDC or cDC-induced proliferation and cytokine production in C57BL/6-derived mediastinal lymph node T cells (CD3+).

**Results:**

Depleting either DC type had modest effect on rejection pathology and T cell proliferation. In contrast, T cells from mice that received grafts depleted of both DCs did not proliferate and this was associated with significantly reduced acute rejection scores compared to all other groups. cDCs were potent inducers of IFNγ, whereas both cDCs and pDCs induced IL-10. Both cell types had variable effects on IL-17A production.

**Conclusion:**

Collectively, the data show that direct allorecognition by donor lung pDCs and cDCs have differential effects on T cell proliferation and cytokine production. Depletion of both donor lung cDC and pDC could prevent the severity of acute rejection episodes.

## Introduction

Lung transplantation is the only therapeutic modality for many patients with end stage lung disease. However, the lung is rejected more often than other grafts and the five year survival is only 50% which is the worst of all solid organs transplants. Many pathologies contribute to the graft dysfunction post transplantation and include primary graft dysfunction (PGD) [1,2], acute rejection and bronchiolitis obliterans syndrome (BO/BOS). All of these are believed to have a component of immune activation resulting in either allo- or autoimmunity that contributes to graft pathology.

Immune activation post lung transplant may be mediated by one of three pathways, direct, indirect or semi-direct [3]. The direct pathway, i.e., mediated by donor derived DCs interacting with recipient T cells, is believed to be the predominant pathway involved in alloimmune activation that leads to rejection in the early post transplant period. Unlike other solid organ allografts, the lung is capable of inducing local alloimmune activation in the absence of any secondary lymphoid organs. Indeed, studies from Kriesel's group demonstrated direct allorecognition occurs in situ within the graft as shown by recipient derived T cells interacting directly with donor derived antigen presenting cells (APCs) leading to activation of allo-reactive T cells [4]. Once activated, recipient lymphocytes may reside within the graft or traffic to regional lymphoid tissues such as the mediastinal lymph nodes [4-7].

While there are multiple types of APCs in the lung, dendritic cells (DCs), the most potent APCs, are believed to have key roles in direct allorecognition post lung transplantation [6,7]. DCs can play a dual role following transplantation; they can induce an alloreactive response against the allograft; or by contrast, they could induce donor-specific tolerance [8]. DCs may be divided into two major subsets, myeloid DCs (cDCs) and plasmacytoid DCs (pDCs), each are known to have specific functions in terms of immune activation. Specifically, cDCs, which are derived from myeloid precursors can drive a potent Th1 immune response [9]. pDCs, on the other hand, which are derived from lymphoid precursors, may have roles in immune regulation, including promoting Th2 driven immune response, as well as IFN-α driven responses [9]. However, the contribution of cDCs and pDCs in allorecognition, in general, as well as post lung transplantation, in particular, are unknown.

In the current study we utilized a BALB/c (H-2^d^) to C57BL/6 (H-2^b^) full MHC mismatch transplant model to assess which APC (cDC or pDC) is responsible for driving the rejection response. Prior to transplantation, we depleted cDCs in the donor lung via a DTR Tg mouse model, or depleted pDCs in the donor lung via a pDCA-1 antibody, or both cDCs and pDCs, followed by an assessment of T cell activation, cytokine networks and rejection pathology.

## Materials and methods

### Animals

Specific pathogen-free male inbred wild-type (Wt) mice BALB/c (H2^d^) and C57BL/6 (H2^b^) were purchased from Harlan Sprague-Dawley (Indianapolis, IN) C.FVB-Tg(Itgax-DTR/EGFP)57Lan/J (CD11c-DTR) mice breeder pairs were purchased from The Jackson Laboratory (Bar Harbor, ME). In these mice, known as DTR Tg, the CD11c promoter is under the control of DTR, and will be referred to as DTR Tg in the current study [10]. The DTR Tg mice were bred onto a BALB/c backcrossing at least eight generations. All studies were done in accordance with institutional guidelines of Laboratory Animal Resource Center at Indiana University School of Medicine. For local DC depletion, Preliminary studies using a range of 10 - 500 ng Diptheria Toxin-DT/mouse injected intratracheally into DTR Tg mice revealed that optimal cDC depletion and minimal toxicity occurred using 50 ng DT/mouse (DT in saline; Sigma-Aldrich, Inc., St. Louis, MO). For local and systemic depletion of pDCs mice were injected intraperitoneally with 500 μg/mouse of anti-mouse PDCA-1 pure functional grade antibody (Ab) clone JF05-1C2.4.1 (Miltenyi Biotech, Auburn CA). All donor and recipient mice were 8-12 weeks of age and 24-32 g.

### Orthotopic lung transplantation

All surgical procedures were performed utilizing sterile techniques. A Prescott's operating microscope (Zeiss 6SFC, Monument, USA) with 20-40× magnification was used for both donor and recipient operations. Both donor and recipient were anesthetized with Isoflurane. The donor→recipient transplanted groups were as follows: Wt BALB/c→Wt C57BL/6; DTR Tg BALB/c→Wt C57BL/6; anti-mPDCA-1 Ab injected Wt BALB/c→Wt C57BL/6; anti-mPDCA-1 Ab injected DTR-Tg BALB/c→Wt C57BL/6. The donor and recipient surgical procedures were performed as previously reported [11]. No mice received any immunosuppressive agents.

### Histology

Mice were euthanized by ketamine (50 mg/kg)/xylazine (10 mg/kg), native and donor lungs harvested, glutaraldehyde-fixed, and paraffin embedded. A portion of the lower lobe of each lung was sectioned and stained with hematoxylin/eosin. Three to six sections from the lower lobes of transplanted lungs were examined and grading of rejection pathology conducted in a blinded fashion utilizing standard criteria for clinical lung transplantation [12]. Scoring was focused on the severity of vascular lesions ("A" scores). A0 = no acute rejection, A1 = minimal acute rejection, A2 = mild acute rejection, A3 = moderate acute rejection, A4 = severe acute rejection.

### Cell Isolation and Characterization

#### T cells

Single cell suspensions were prepared from the mediastinal lymph nodes of transplant recipient mice. T cells were isolated using CD90.2 (Thy1.2)^+ ^Microbeads (Miltenyi Biotech, Auburn CA) according manufacturer's instructions and were > 98% CD3^+ ^as reported [13].

### Plasmacytoid and Myeloid Dendritic cells

Single cell suspensions were prepared from the lungs of wild-type BALB/c mice. Plasmacytoid DCs cells were isolated using anti-mPDCA-1 microbeads and cDCs were isolated using CD11c microbeads (Miltenyi Biotech, Auburn CA) according manufacturer's instructions.

### Mixed Leukocyte Reaction (MLR)

Mixed leukocyte reactions were conducted by culturing irradiated stimulator cells [(3 × 10^4^) Wt BALB/c cDC or pDC] in the in the presence of responder CD3^+ ^T cells isolated from the mediastinal lymph nodes of C57BL/6 transplant recipient mice (1 × 10^6^/ml) in 96-well plates. Cultures were pulsed after 48 hr with 1 μCi of [H^3^] thymidine, and of [H^3^] thymidine incorporation was measured 18 hr later.

### Cytokine profiling by cytometric bead array (CBA)

Culture supernatants from allograft transplant C57BL/6 recipients were collected and cytokine protein levels of IL-17A, IL-10, TNF-α, IFN-γ, IL-6, and IL-4 were measured using the Mouse Th1/Th2/Th17 Cytokine Kit (BD Biosciences, San Jose, CA) according to manufacturer's instructions.

### Flow cytometry

All staining reactions were performed at 4°C and 2.4G2 Fc receptor Ab (CD16/CD32) was added to reduce nonspecific binding. Lung mononuclear cells were obtained by homogenizing lungs using the gentle MACS Dissociator (Miltenyi, Auburn, CA) followed by Percoll gradient purification. The mononuclear cells were stained for APC subsets using F4/80-APC Cy7 (BM8; Biolegend), PDCA-1-Alexa Fluor 647 (eBio927; eBioscience), CD11b-PerCPCy5.5 (M1/70; BD Biosciences), CD11c-PE Cy7 (HL3; BD Biosciences), B220-PE Cy5 (RA3-6B2; BD Biosciences), MHC class II-PE (M5/114; eBioscience) and Gr1-PE (RB6-8C5, BD Biosciences). Cells were fixed using 1% paraformaldehyde and analyzed using a BD LSRII Flow Cytometer System. cDCs were defined by CD11c^+^, CD11b^low^, MHC class II^+^, B220^-^, Gr1^-^. pDCs were defined as PDCA^+^, CD11c^low^, B220^+^, Gr1^-^, MHC class II^+^.

### Statistical analysis

Statistical analysis was conducted using one-way ANOVA test and posthoc comparisons using Tukey's test for multiple comparisons. Data expressed as means ± Standard Deviation. A "p value" of < 0.05 was considered significant. All analyses were performed using a statistics software package (GraphPad Prism 4).

## Results

### Donor lung cDC or pDC depletion prior to transplantation

**Figure 1:**
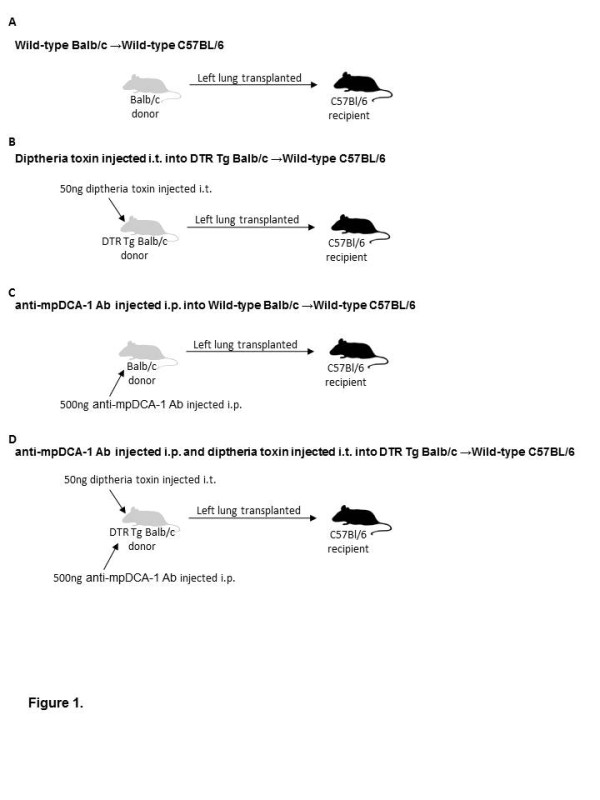
**cDC and/or pDC depletion setup in murine orthotopic transplant model**. Donor →recipient transplanted groups: Wt BALB/c→Wt C57BL/6; DT (50 ng) injected DTR-Tg BALB/c→Wt C57BL/6; anti-mPDCA-1 Ab (500 ng) injected Wt BALB/c→Wt C57BL/6; anti-mPDCA-1 Ab (500 ug) and DT (50 ug) injected DTR-Tg BALB/cc→Wt C57BL/6. N = four to six mice per group.

With previous reports suggesting that the initiation of lung rejection may be due to T cell priming in the lung allograft following transplantation, and with evidence demonstrating the involvement of dendritic cells (cDCs and pDCs) in allorecognition, we determined the role of each DC subset in stimulating alloimmune responses in T cells. To address this we utilized a full-MHC mismatch orthotopic transplant model, in which a BALB/c (H-2^d^) donor lung was transplanted into a C57BL/6 recipient (H-2^b ^[Figure 1A]. This model was chosen due to the reproducible and robust development of severe acute rejection (ISHLT Grade A4) within seven days post transplantation [14]. To examine the role of each cell subset, we depleted cDCs or pDCs or both cell types in donor mice prior to harvesting lungs for transplantation. cDCs were depleted using a DTR Tg (BALB/c) mouse model, in which 50 ng of diphtheria toxin (DT) was delivered via intratracheal instillation (i.t.) [Figure 1B]. Two days later, the time at maximal cDC depletion, the left lung was used as a donor lung and transplanted into a wildytpe C57Bl/6 mouse. Seven days later, the mediastinal lymph nodes from the transplant recipient were harvested and CD3^+ ^T cells were isolated and cultured. In complementary studies, pDCs were depleted using an antibody approach, in which a BALB/c mouse was injected intraperitoneally with 500 μg of anti-mPDCA-1 Ab [Figure 1C] as reported [15]. Three days later, the time to maximal pDC depletion, the left lung was transplanted into a wildtype C57BL/6 mouse. Seven days following, the mediastinal lymph nodes were harvested from the transplant recipient and CD3^+ ^T cells were isolated and cultured. To deplete both subsets, the DTR Tg mice were utilized as the donor and were given an i.p. injection of 500 μg of anti-mPDCA-1 Ab 3 days prior to transplantation and 50 ng of DT was given two days prior to transplantation [Figure 1D]. Again, seven days later the mediastinal lymph nodes were harvested and CD3^+ ^T cells were isolated and cultured. In all conditions, the transplant recipient was a C57Bl/6 mouse from which mediastinal lymph node T cells were purified (CD3^+^). pDCs and cDCs utilized for restimulating T cells ex-vivo were always isolated from the lungs of normal BALB/c mice and therefore were derived from the same strain of mice in which DC depletion occurred.

**Figure 2:**
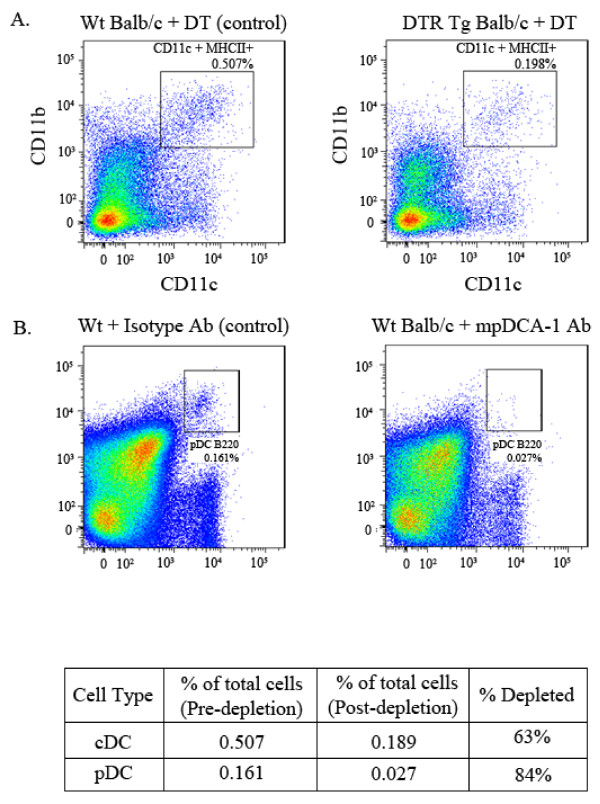
**Verification of cDC or pDC depletion by flow cytometry**. Lung mononuclear cells were stained for DC subset markers. Cells are reported as percent of total population. Macrophages were excluded using FL1 autofluorescence and the macrophage specific marker F4/80. A) Myeloid dendritic cells: B220^-^, Gr1^-^, CD11b^+ ^and CD11c^+^. Wt BALB/c + DT (non-depleted control) vs. DTR Tg BALB/c + DT (depleted) yield a 63% depletion. B) Plasmacytoid dendritic cells: CD11clow, B220^+^, and PDCA^+^. Wt + isotype Ab (non-depleted control) vs. Wt Balb/c + mPDCA-1 Ab (depleted) yields a 84% depletion. Data are representative of four to six mice in each group.

To verify that cDCs were depleted in the DT treated DTR Tg donor lung, mononuclear cells were isolated from the donor treated lungs and assessed for cDCs by flow cytometry as described in Methods. As shown in Figure 2A, compared to DT treated Wt BALB/c donor lungs, a representative DT treated DTR Tg donor lung showed a 63% reduction in the cDC population, thereby demonstrating cDC depletion in the donor lung. It is important to note that in addition to depleting cDCs, in this DTR Tg mouse model, alveolar macrophages are also depleted by roughly 50%. F480 staining was utilized to monitor macrophage depletion during these studies. To verify pDC depletion in the anti-mPDCA-1 Ab treated donor lung, lung mononuclear cells were isolated from the donor treated lungs and assessed for pDCs as described in Methods. As shown in Figure 2B, compared to isotype (rat IgG2b) treated Wt BALB/c donor lungs, mPDCA-1 Ab treated wildtype BALB/c donor lungs showed a nearly 84% reduction in the pDC population in a representative lung, thereby demonstrating pDC depletion in the donor lung.

### T cell alloreactivity in response to cDC or pDC stimulation

**Figure 3:**
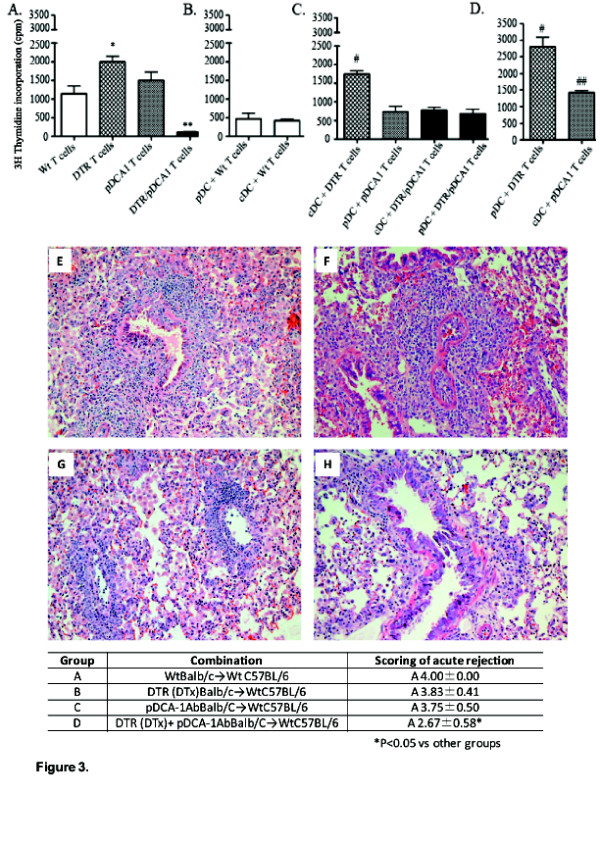
**T cell alloreactivity in response to cDC or pDC stimulation**. Lung transplantation was performed as described in Methods and transplanted lungs harvested at 7 days post transplant. A-D) Mediastinal lymph node CD3+ T cells from lung transplant recipient (C57BL/6) mice were cultured alone (3 × 10^5^) or in the presence of Wt donor (BALB/c)-derived cDCs or pDCs (3 × 10^4^). Cells were incubated for 72 h at 37°C. T-cell proliferation was measured by 3H thymidine incorporation. A) Spontaneous T cell proliferation of Wt, DTR, PDCA1 and DTR/pDCA1 transplant groups. B) cDC and pDC were co-cultured with Wt T cells isolated from lung transplant recipients that received dendritic cell sufficient lung allografts. C) cDC and pDC co-cultured with recipient-derived T cells isolated from cDC, pDC or cDC/pDC depleted transplant groups. D) pDC and cDC co-cultured with recipient-derived T cells isolated from cDC and pDC depleted transplant groups. Responses of recipient cells were compared using one-way ANOVA analysis. ** p < 0.01, # p = 0.01, ## p = 0.02 * p < 0.05 Panels E-H represent H&E stained lung sections from allografts harvested at day 7. Table 2) Histologic rejection scores for transplant groups. Data represent the mean +/- SD of "A" scores for four to six mice in each group harvested at 7 days. *p < 0.05 compared to other groups. (40× magnification).

It is unclear as to which DC subset drives the alloresponse and which DC is important in inducing T cells proliferation. With verification of cDC or pDC depletion in the donor lung prior to transplantation, we next examined spontaneous T cell proliferation from untreated Wt allograft recipients, cDC depleted allograft recipients, pDC depleted allograft recipients or double depleted (cDC and pDC) allograft recipients. Figure 3A represents the culture of transplant recipient CD3^+ ^T cells alone, in the absence of any stimulation (cDCs or pDCs). As shown in Figure 3A, T cells from the wild-type allograft (Wt T) recipient [white bar] spontaneously proliferate. Additionally, T cells from the DTR Tg allograft (DTR T) recipient [hatched bar], and T cells from the mPDCA-1 Ab treated allograft (pDCA1 T) recipient [gray bar] both spontaneously proliferate, with the DTR T proliferating significantly more than the Wt T cells (p < 0.05). Interestingly, T cells from the double depleted allograft (DTR/pDCA1 T) recipient [black bar] did not spontaneously proliferate, as compared to the Wt T cells.

Next, we examined the alloresponsiveness of T cells from the allograft recipient (C57BL/6-derived) following restimulation with BALB/c mouse-derived cDCs or pDCs. These studies would allow for determining which donor lung DC subset had key roles in priming of recipient T cells. To assess this, allograft recipient mediastinal lymph node CD3^+ ^T cells were co-cultured with lung-derived cDCs or pDCs. Due to the limitations in acquiring large enough numbers of cDCs, and pDCs from the transplanted mice for the purposes of multiple co-cultures with the CD3^+ ^recipient T cells, we chose take a different approach. In each co-culture condition, wild-type lung-derived BALB/c cDCs or pDCs were isolated and co-cultured with the various transplant recipient CD3^+ ^mediastinal lymph node T cells; T cell proliferation was then assessed [Figure 3B-D].

In panel B of Figure 3, we examined the response of Wt T cells to cDCs or pDCs. As compared to the spontaneous proliferation of the Wt T cells alone, when BALB/c-derived cDCs or pDCs were co-cultured with Wt T cells from normal C57BL/6 mice, the cells did not proliferate briskly. Moreover, when recipient T cells from the DT treated DTR allograft (cDC depleted) were restimulated with BALB/c-derived cDCs, there was no significant change in proliferation as compared to T cells alone [Figure 3C, hatched bar]. Interestingly, when recipient T cells from mPDCA-1 treated allograft (pDC depleted) were restimulated with BALB/c-derived pDCs, they proliferated less, as compared to the spontaneously T cells from pDCA1-treated mice [Figure 3C, gray bar]. When BALB/c-derived cDCs or pDCs were used to restimulate recipient T cells from the double DC-depleted allograft recipients, there was a significant increase in proliferation as compared to unstimulated T cells alone [Figure 3C, black bars]. When BALB/c-derived pDCs were used to restimulate T cells (C57BL/6) from DT treated DTR mice lung transplant recipients, there was a significant increase in proliferation compared to the spontaneously proliferating DTR T cells [Figure 3D, hatched bar]. Lastly, when T cells from pDC-depleted lung transplant recipients were restimulated with BALB/c cDCs, there was no change in proliferation when compared to the spontaneously proliferating pDCA1 T cells [Figure 3D, gray bar]. Collectively, these data suggest that both cDC and pDC in the donor lung contribute to alloantigen-induced priming of recipient T cells. However, donor lung-derived pDCs appear to more potent than cDCs in alloantigen-induce priming post lung transplantation.

Lung allograft rejection is believed to be initiated by donor derived lung DCs activating recipient T cells. Data showing that lymph node T cells from mice that had lungs depleted of both cDCs and pDCs did not spontaneously proliferate (Figure 3A) could suggest that depletion of both cDCs, pDCs, or both may prevent or down regulate rejection pathology. Using standard rejection pathology criteria [12] we examined the histology in the BALB/c→C57BL/6 full mismatch model. All untreated transplanted lungs had undergone severe acute rejection (Grade A4) within seven days post transplantation [Figure 3E]. cDC depletion in the allograft lung, DTR(DT) BALB/c→C57BL/6, slightly decreased acute rejection [Figure 3F]. pDC depletion in the allograft lung, mPDCA-1 Ab BALB/c→C57BL/6, resulted in a similar decrease in acute rejection compared to cDC depletion [Figure 3G]. However, double depletion, DTR/mPDCA1, significantly decreased acute rejection even further as compared to all other groups [Figure 3H] (p < 0.05).

### cDC or pDC-induced cytokine production

**Figure 4:**
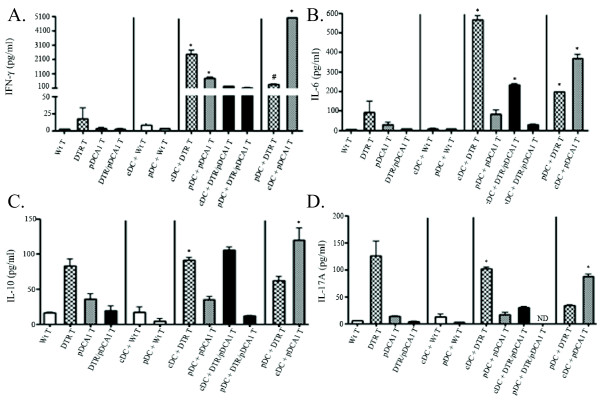
**Cytokine production in response to cDC or pDC stimulation**. Mediastinal lymph node CD3^+ ^T cells from lung transplant recipient (C57BL/6) mice were cultured alone (3 × 10^5^) or in the presence of Wt donor (BALB/c)-derived cDCs or pDCs (3 × 10^4^). Cells were incubated for 72 h at 37°C. Culture supernatant was collected and A) IFN-γ, B) IL-6, C) IL-10, and D) IL-17A were measured by cytometric bead assay. * p < 0.01, # p < 0.05 compared to cDC + Wt T and pDC + Wt T groups by one-way ANOVA with Dunnett's Multiple Comparison post-test.

With data demonstrating that pDCs may be important for initiating the alloimmune response, we next determined spontaneous, pDC or cDC-induced cytokine production-derived from mediastinal lymph node T cells isolated from lung transplant recipients. Cytokine profiles were assessed within the supernatants from the co-culture experiments shown in Figure 3. Figure 4 data is shown to match the groups show in the proliferation studies of Figure 3. Spontaneously proliferating recipient T cells from each condition secreted low or non-detectible levels of IFN-Y, and neither BALB/c cDC nor pDC induced IFN-Y from wildtype T cells (CD3+) isolated from normal C57BL/6 mice [Figure 4A]. Notably, recipient T cells produced IFN-γ when either cDC, pDCs or both were depleted from the graft prior to transplantation (Figure 4A). However, cDCs appear to have a major role in IFNγ induction since IFN-γ responses were most robust in T cells isolated from mice that received lungs depleted of pDCs and restimulated with cDCs (Figure 4A). These data suggest a role for donor lung-derived cDC in stimulating IFN-γ production. These data are consistent with the known role of cDCs in the induction of Th1 (IFN) responses [16].

T cell cultures revealed low level IL-6 production, constitutively. Since IL-6 is made by mononuclear cells, other than T cells, then this production could have been due to the presence of non-T cells in the cultures (Figure 4B). However, T cells may induce DCs to produce IL-6. Interestingly, very low levels of IL-6 were detected in the co-cultures of cDCs or pDCs with wildtype lymph node T cells [Figure 4B]. In contrast, IL-6 was detected in co-cultures in which T cells had been primed with cDCs, pDCs, or severely reduced populations of both cDC and pDC (cDC + DTR T, cDC + pDCA T, or cDC + DTR/pDC T groups) (Figure 4B).

Depletion of cDCs resulted in significantly greater spontaneous IL-10 production from recipient T cells (DTR T group) as compared to wildtype T cells or T cells isolated from lung allograft recipients depleted of pDCs or both cDCs and PDCs (Figure 4C), These data could suggest pDC may have a role in stimulating IL-10 production from recipient T cells, and could be consistent with reports demonstrating that pDCs can promote regulatory T cell (Treg) development, which is in part characterized by expression of CD4+Foxp3+ in T cells [17]. However, the highest level of IL-10 was induced by cDCs in co-culture with T cells from pDC-depleted lung allograft recipients (cDC + pDCA T). Furthermore, pDCs and cDCs appear comparable in stimulating IL-10 production from mice that received lungs deficient in cDCs (cDC + DTR T, and pDC + DTR T groups), [Figure 4C]. Collectively, these data suggest a complex relationship of cDC or pDC-alloantigen induced IL-10 production from recipient T cells.

Similar to IL-10, depletion of cDCs resulted in significantly greater spontaneous IL-17A production from recipient T cells (DTR T group) as compared to wildtype T cells or T cells isolated from lung allograft recipients depleted of pDCs or both cDCs and PDCs [Figure 4D]. In general, the production of IL-17A from T cells [Figure 4D] followed the same patterns as shown for IL-10 [Figure 4C]. One exception is data showing that pDCs, in contrast to cDCs, were not able to induce any IL-17A production from mice that received lungs deficient in both cDC and pDCs. These data could also be explained by pDC-induced suppression of IL-17A production. Neither TNF-α nor IL-4 were detected at significant levels in any assay condition (data not shown).

## Discussion

Dendritic cells (DC) are the most potent APCs in regulating adaptive immune responses. As such, DCs have been reported to have important/dual roles in regulating alloimmunity leading to either transplant rejection or tolerance. Since lung allograft rejection is thought to be initiated by donor derived lung DCs activating recipient T cells, we examined the role of donor-derived DC subsets (cDCs and pDCs) in stimulating the responsiveness of recipient T cells. In this study we demonstrated that depletion of both donor allograft cDCs plus pDCs down regulated the pathology of lung allograft rejection. Furthermore, T cells isolated from mice that received lungs depleted of both cDCs and pDCs did not spontaneously proliferate, as compared to T cells from mice that received grafts depleted of either cDC or pDCs. In our assessment of T cell priming, we demonstrated that although both cDC and pDC in the donor lung contribute to alloantigen-induced priming of recipient T cells, donor lung-derived pDCs appear to be more potent than cDCs in alloantigen-induced proliferation, whereas donor lung-derived cDCs were potent inducers of IFN-γ responses.

Maturation status has been linked to the ability of DCs to induce or suppress immune responses. Specifically, immature DCs may be suppressive/tolerogenic whereas mature DCs may be highly immunogenic [16]. However, DC immunogenicity may also be linked to specific subsets. cDCs and PDCs comprise two major groups of DCs in all tissues [18]. In the context of organ transplantation, cDCs are reported to be highly immunogenic and efficient in stimulation anti-T cell alloimmunity, including production of IFN-γ [19]. In contrast, pDCs have been reported to be tolerogenic [20]. Specifically, suppression of alloreactive T cell via induction of regulatory T cells or clonal deletion of alloreactive T cells have been functions ascribed to pDCs [21]. pDCs may be responsible for suppressing activation and proliferation of alloreactive T cells via induction of T regulatory cells (Tregs), inhibiting memory T cell responses or clonal deletion, thereby promoting tolerance and preventing graft rejection in non-pulmonary allografts [14-16,22-24]. Consistent with prior reports in other organs, the current study shows that lung cDCs are potent inducers of IFN-γ production, and contribute to induction of IL-10. However, pDCs did not appear to be tolerogenic as shown by studies in which cDC depletion did not lead to improved outcomes histologically. Abe et al., reported that pDCs propagated from donor or third-party bone marrow could prolong murine cardiac allograft survival [22]. In another study, host-derived pDCs played an important role in development of allograft tolerance. In this study, host-derived pDCs acquired donor antigens from the allograft and migrated to the lymph node, where they induced alloantigen-specific Tregs, which lead to the induction of tolerance. Depletion of pDCs in this model, inhibited Treg development and the induction of tolerance [24]. Similar findings have been reported in human liver transplant studies [17]. In contrast, depletion of both cDC and pDC resulted in diminished spontaneous T cell proliferation and prevented rejection pathology. These data highlight a role for both lung cDCs and pDCs in initiating allo-responsiveness post lung transplantation. The differences in the potential roles of pDCs in tolerance and immunogenicity in the current study could be related to the fact that lung-derived DCs are phenotypically and functionally distinct from other tissue-derived DCs [25]. Accordingly, data derived from a non-pulmonary transplant models are not likely to be extrapolated to, or reproduced in the lung.

In our model, we utilized naïve cDCs and pDCs as stimulators in the presence of recipient T cells. Since cDCs and pDCs make up less than 1% of the total cell population in the lung, we were not able to isolate sufficient allograft cDCs or pDCs to use for ex vivo stimulation. Doing so would have allowed us to examine the function of DCs conditioned by the allograft environment in stimulating T cells. However, this assessment would have required transplanting and harvesting more donor and recipient allografts than could be performed in an individual experiment. Another aspect that could have impacted our results examining T cell activation is effect of donor lung-derived non-professional APCs, such as epithelial [26,27] and endothelial [28] cells in stimulating T cells.

## Conclusion

These data highlight the importance of both DC subsets in initiating alloimmune responses post lung transplantation. A limitation of the data presented is that there were residual cDC and pDC in depleted mice as has been reported by other using these techniques [10]. Accordingly, some observed effects could have been to due residual cells of each type in treated mice, or possibly due to effects mediated by recipient DCs [29]. Although it has been thought that pDCs are required to promote tolerance and prolong allograft survival, it appears that the absence of both cell subsets contribute to alloimmune activation. Therefore, it is interesting to speculate that depletion of both pre-transplant could lead to diminished acute rejection episodes.

## Competing interests

DSW is co-founder and Chief Scientific Officer of ImmuneWorks Inc., a biotech company developing novel therapeutics for immune mediated lung diseases. No other authors have any conflicts to disclose and no part of this work was supported by a commercial organization, including ImmuneWorks.

## Authors' contributions

Note HLB and HS are co-first authors due to equal contributions

HLB prepared the manuscript and conducted in vivo and in vitro experiments. HS prepared the manuscript and conducted lung transplant surgeries. JL performed cell isolation and proliferation assays. AF assisted will tissue harvest post transplantation. CW conducted flow cytometry for dendritic cells. KH assisted HB, AJ and AF. RB bred the DTR Tg mice. JSB provided expertise in dendritic cell isolation. DSW conceived the study and developed overall experimental design, and manuscript preparation. All authors read and approved the final manuscript.
